# The Mammalian Circadian Time-keeping System

**DOI:** 10.3233/JHD-230571

**Published:** 2023-04-25

**Authors:** Andrew P. Patton, Michael H. Hastings

**Affiliations:** Medical Research Council Laboratory of Molecular Biology. Francis Crick Ave, Cambridge, CB2 0QH, U.K., Tel: 00 44 1223 267045

**Keywords:** Astrocyte, Cryptochrome, Feedback Loop, Hypothalamus, Melanopsin, Neuropeptide, Neurodegeneration, Period, Retina, Suprachiasmatic Nucleus

## Abstract

Our physiology and behaviour follow precise daily programmes that adapt us to the alternating opportunities and challenges of day and night. Under experimental isolation, these rhythms persist with a period of approximately one day (circadian), demonstrating their control by an internal autonomous clock. Circadian time is created at the cellular level by a transcriptional/translational feedback loop (TTFL) in which the protein products of the *Period* and *Cryptochrome* genes inhibit their own transcription. Because the accumulation of protein is slow and delayed, the system oscillates spontaneously with a period of ~24 hours. This cell-autonomous TTFL controls cycles of gene expression in all major tissues and these cycles underpin our daily metabolic programmes. In turn, our innumerable cellular clocks are co-ordinated by a central pacemaker, the suprachiasmatic nucleus (SCN) of the hypothalamus. When isolated in slice culture, the SCN TTFL and its dependent cycles of neural activity persist indefinitely, operating as “a clock in a dish”. In vivo, SCN time is synchronised to solar time by direct innervation from specialised retinal photoreceptors. In turn, the precise circadian cycle of action potential firing signals SCN-generated time to hypothalamic and brain stem targets, which co-ordinate downstream autonomic, endocrine and behavioural (feeding) cues to synchronise and sustain the distributed cellular clock network. Circadian time therefore pervades every level of biological organisation, from molecules to society. Understanding its mechanisms offers important opportunities to mitigate the consequences of circadian disruption, so prevalent in modern societies, that arise from shiftwork, ageing and neurodegenerative diseases, not least Huntington’s disease.

## Introduction

The alternation between day and night presents us with a regular and predictable series of environmental challenges and opportunities. To adapt to this, evolution has furnished us with daily rhythms of physiology and behaviour, the most obvious being the sleep/wake cycle (SWC) that sustains energetic daytime engagement with the world and night-time withdrawal to allow rest, growth and repair. Underlying and supporting the SWC, almost all aspects of our metabolism, brain function, endocrine and autonomic states progress through a regular daily programme. In essence, our body is a 24-hour machine and what it is pre-programmed to do in the day is very different from its capabilities at night [1]. An important observation is that even though our daily rhythms are ordinarily synchronised to the light/dark cycle, they are not caused by it. Rather, when humans and experimental animals are isolated in a time-free environment of constant light, temperature etc., the SWC and attendant rhythms continue to run with a period of approximately one day (hence, *circadian*). This autonomy demonstrates that these rhythms are driven by an internal timing system, a biological clock [2]. Nevertheless, such pervasive internal daily patterning is commonly overlooked unless and until it goes wrong, as can occur, for example, in sleep-disorders [3], shift-work [4], ageing and neurodegenerative disease [5, 6]. The purpose of this review is to consider the molecular, cellular and physiological basis of the circadian clock of mammals and how it orchestrates our temporal adaptation to the world.

### 1 Daily regulation of physiology and behaviour: clocks and sleep

**Figure 1:**
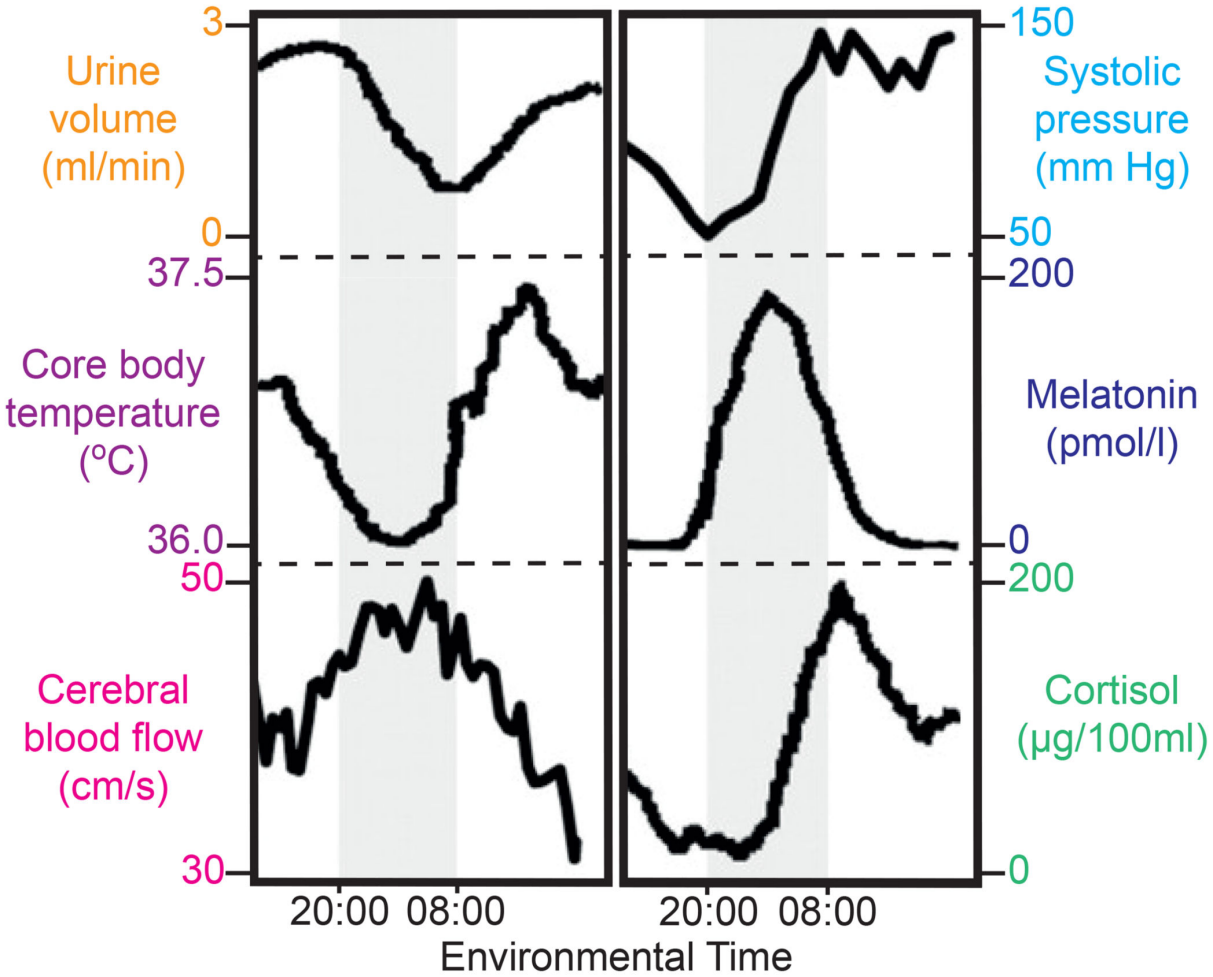
The human circadian programme. Schematic representation of circadian variations of physiological and endocrine status recorded in human volunteers subject to a constant routine protocol. Grey shading represents expected sleep interval, which was denied by constant routine protocol. Based on [2].

The extent and persistence of circadian regulation in humans is apparent when volunteers are subject to experimental isolation for several weeks. Accompanying the SWC are, for example, circadian rhythms in circulating melatonin, a marker of circadian night, and cortisol, which peaks at the start of biological day to prepare the body for wakefulness and physical activity. Alongside this, rhythms of core body temperature and heart rate reflect circadian control of autonomic function. It could be suspected that these changes are secondary to the SWC and the associated circadian patterns of eating and drinking, but that is not the case. In Constant Routine studies, subjects are kept continuously awake in bed with a constant posture, continuous exposure to dim light, and small meals are taken evenly throughout the protocol [1]. Although this may reduce the amplitude of some of the rhythms, betraying an influence of sleep and wakefulness, their periodicity continues unabated, highlighting their direct control by the circadian system (Figure 1). By removing any temporal pattern to ingestion with the Constant Routine, circadian control of circulating levels of many key metabolites can be seen, indicative of underlying rhythms in hepatic and other organ systems [7]. Furthermore, the constant wakefulness of this routine also reveals circadian control of brain function, as evidenced by rhythms in performance on psychomotor vigilance and memory tests.

The interplay between the circadian clock and sleep in sculpting daily rhythms can be explored further by Forced Desynchrony protocols, in which subjects are kept on an imposed SWC cycle longer or shorter than 24 hours [8]. The circadian system is unable to synchronise to such a regime and so its physiological outputs, such as the rhythm of melatonin, free run with their intrinsic clock-controlled period that is stable for any individual and, across a healthy population, ranges between 23.5 and 24.7 hours [9]. Consequently, over the course of several weeks, the imposed bouts of sleep and wakefulness “scan” across all circadian phases, and vice versa. This has shown that sleep efficiency is greatest when it coincides with circadian biological night, and least when it is mis-aligned by the protocol to fall in circadian daytime. Furthermore, exogenous melatonin administered in circadian daytime can enhance the quality of such mis-timed sleep, emphasising the value of temporal alignment between components of the circadian system in directing physiology [1]. In a similar manner, the Forced Desynchrony protocol has also demonstrated that psychomotor and cognitive performance are determined by both circadian phase and time spent awake. Under normal circumstances these factors operate in unison to facilitate performance, but their mis-alignment carries a negative impact. More generally, it is not surprising, therefore, that mis-alignment of sleep and circadian systems carries significant costs for metabolic [10, 11] and mental health [12–14].

Studies in experimental animals, particularly mice, provide a molecular genetic perspective on the circadian programmes they share with humans. With the advent of DNA micro-arrays and, more recently, RNA sequencing (RNAseq), it is clear that between 10% and 20% of the genes expressed in peripheral tissues such as liver, kidney and skeletal muscle are under tight circadian regulation [15–18]. Many of these genes encode proteins that are rate-limiting in metabolic pathways and essential for life [19, 20], and yet their transactivation only occurs for limited part of the circadian cycle, emphasising the precision and accuracy of the circadian regulation of metabolism. The mapping of gene expression across multiple mouse tissues reveals that over 40% of all protein-coding genes exhibit organ-specific circadian modulation [21]. Furthermore, the temporal organisation of tissue-specific gene transcription declines with mouse age [22, 23], which in turn may be a cause of age-related morbidity. Available evidence suggests a comparably extensive circadian transcriptional programme exists across all major organ systems, including brain regions, in non-human primates [24], albeit with some differences attributable to the effects of diurnal versus nocturnal habits. Although such comprehensive analysis is difficult in humans, circadian control over ~6% of the blood transcriptome has been demonstrated under a Constant Routine, with different transcripts exhibiting day- or night-specific peaks of expression [25]. Even more striking, under a Forced Desynchrony protocol, this fell to ~1% of expressed transcripts, highlighting the importance of internal temporal alignment for metabolic function.

It is likely, therefore, that elucidating the mechanisms active in experimental animals will have direct relevance to the human system. Indeed, *“…the majority of best-selling drugs and World Health Organization essential medicines directly target the products of rhythmic genes…”* [21]. Given the rapid pharmacokinetics of such drugs, their clinical efficacy may, therefore, be improved by considering the timing of dosage to match target vulnerability. Similarly, the tight interplay between clocks and metabolism revealed experimentally in mice [26] [27] and the epidemiological evidence of poor metabolic heath evident with modern life-styles [28], suggests circadian-based approaches could be helpful to treat diseases characterised by metabolic disruption [29]. More broadly, it has been argued that circadian time should be factored into our approaches to diagnosis, management and therapy [30, 31]. Circadian time-keeping is not only biologically fascinating, but its application also holds enormous clinical relevance.

### 2 The suprachiasmatic nucleus as circadian pacemaker

**Figure 2:**
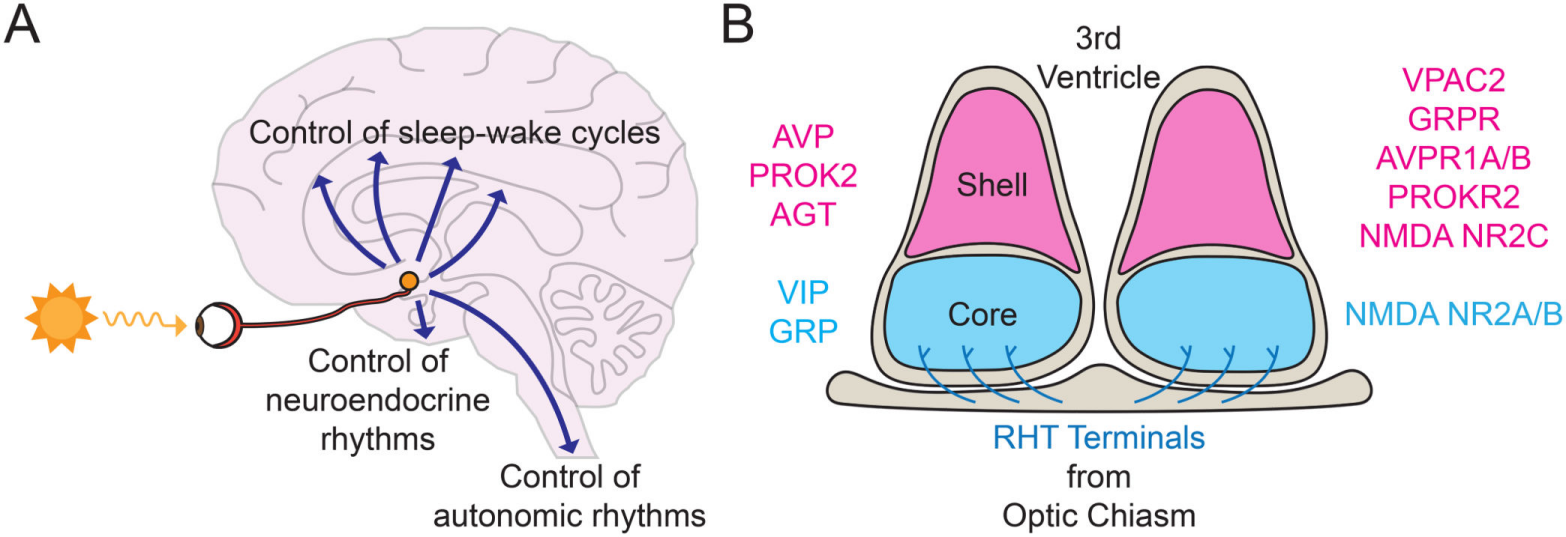
The suprachiasmatic nucleus (SCN) as central circadian pacemaker. A. Schematic sagittal view of the location of the human SCN, at the base of the hypothalamus, depicting retinal input from the retinohypothalamic tract (RHT) via the optic nerve, and outputs to neural centres controlling sleep-wake behaviour, neuroendocrine and autonomic status. B. Schematic coronal view of mouse SCN to show ventral retinorecipient core and dorsal shell sub-divisions, characterised by their distinction expression of (left) neuropeptides and (right) neurotransmitter receptors, and RHT innervation of SCN core.

So where is the clock that controls our daily life? Circadian rhythms are synchronised to the light-dark by entraining cues delivered from the retina: experimental rodents [32, 33] and people [34] lacking a functional retina fail to entrain to their daily lighting cycle and instead their rhythms free-run at their intrinsic circadian period. The mapping of retinal projections to the hypothalamus, which was already known from lesion studies to direct circadian behaviour, revealed the suprachiasmatic nucleus (SCN) as a candidate photoentrainable clock [32] (Figure 2A). Subsequent lesion studies in rodents showed that an intact SCN is necessary to sustain circadian behaviour and endocrine rhythms in experimental rodents [35, 36], whilst damage to the SCN in humans that may arise from compression by pituitary tumours [37] or aneurysm [38] has been associated with altered quality and timing the SWC. Of course, loss-of-function does not prove the SCN is the clock: it could alternatively be an essential link in an output pathway conveying time cues from a clock elsewhere to the rest of the brain. The definitive test of the SCN as the principal clock in mammals came from a series of studies showing, first, that the SCN is autonomously rhythmic, with circadian rhythms of electrical firing and metabolism both in vivo and ex vivo, peaking in circadian day. Second, that manipulations of SCN activity change circadian behaviour and that genes that alter circadian period in vivo also alter the period of the SCN. Finally, grafting of the SCN into arrhythmic SCN-lesioned rodents restored circadian behaviour in the recipients and, more importantly, the behavioural period was specified by the genotype of the grafted tissue rather than that of the host animal [39].

Thus, the SCN is necessary and sufficient for the circadian control of behaviour. In some ways this is remarkable insofar as each SCN flanking the third ventricle and sitting above the optic chiasm, consists only of ~10,000 neurons and ~3,000 glial cells [40]. And yet its influence is so pervasive and sustained throughout life. The neurons are segregated into two principal divisions (Figure 2B). The ventral core receives direct retinal afferents by way of the retinohypothalamic tract (RHT), and contains neurons that express the neuropeptides vasoactive intestinal peptide (VIP) and gastrin-releasing peptide (GRP). The dorsal shell does not receive a strong retinal innervation, and contains neurons that express arginine vasopressin (AVP), prokineticin 2 (PROK2) and angiotensin, as well as receptors for VIP, GRP, AVP and PROK2 [40, 41]. Both core and shell give rise to efferent projections that run to adjacent areas of the hypothalamus, the midline thalamus and the brainstem, thereby providing access to centres that control autonomic, endocrine and behavioural (wake/arousal) states [42, 43]. The advent of single-cell RNA sequencing has made it possible to reveal with greater resolution the transcriptional diversity of cell types in the mouse SCN and to assign a more sophisticated topology to its intrinsic circuitry beyond the core-shell dichotomy [44, 45] and this complexity may be related to the robust circuit-level time-keeping of the SCN [46] (see below). An important general principle, however, is that the cellular phenotypes and patterns of connectivity of the SCN observed in rodents are conserved across primates and humans [47–49].

To be adaptive, the SCN clock and its rhythm of electrical activity has to be entrained to, and therefore predictive of, solar time. This entrainment is mediated by RHT afferents that enter the SCN core from the optic chiasm and release the excitatory neurotransmitter glutamate (Glu) in response to illumination of the retina. The retinorecipient SCN neurons are activated through their glutamatergic ionotropic receptors, initially being depolarised by rapidly acting AMPA-type receptors, which in turn facilitate subsequent activation of NMDA-type receptors which are permeable to Ca^2+^ ions. Depolarisation and enhanced electrical firing are therefore accompanied by Ca^2+^-dependent gene expression. Light-induced activation of electrical firing of retinorecipient neurons at dusk delays their ongoing drift to quiescence, whereas light presented around dawn advances the programmed onset of their electrical activity [50]. These shifts of the SCN electrical activity cycle cause its intrinsic ~24 hours period to be lengthened or shortened to exactly 24 hours by dusk and dawn light, respectively. Indeed, the circadian behaviour of experimental animals and people can be stably entrained to 24 hours by “skeleton” photoperiods in which light is only presented at dawn and dusk. The size of the individual phase-shifts is determined by the intensity and duration of the exposure to light in an integrative manner of “photon counting”. This is again a general property of entrainment that is shared between humans and other mammals [51–53]. The outcome of light-dependent resetting of the SCN is to hold it in an appropriate phase relationship to the cycle of light and darkness, with electrical and metabolic activity peaking in day and being quiescent at night. This is the case in both nocturnal and diurnal species: the SCN encodes solar time, not behavioural habit [54].

The RHT axons arise from a sub-population of retinal ganglion cells (RGCs) that are intrinsically photoreceptive (ipRGCs) [55], and so entrainment of the SCN and circadian behaviour does not require functional rods and cones [56]. The discovery in mammals of a novel opsin, melanopsin, as an inner retinal photopigment expressed in ipRGCs, provided a completely new perspective on circadian entrainment [57]. In contrast to rods and cones, which hyper-polarise in light, melanopsin-expressing ipRGCs are depolarised by light, firing action potentials at a sustained higher frequency with brighter illumination [58]. Hence, they can integrate luminance over long time-frames, making them ideal for the “photon counting” of circadian entrainment, and acting independently of image-forming circuits in the brain. Furthermore, rodent studies have revealed that other projections from ipRGCs to midline thalamus, hypothalamus and brain stem mediate the effects of lighting on mood states and alertness [59], and the available evidence indicates that the ipRGC system is an equally important component of subliminal responses to light in humans [60].

### 3 The cell-autonomous clock

**Figure 3:**
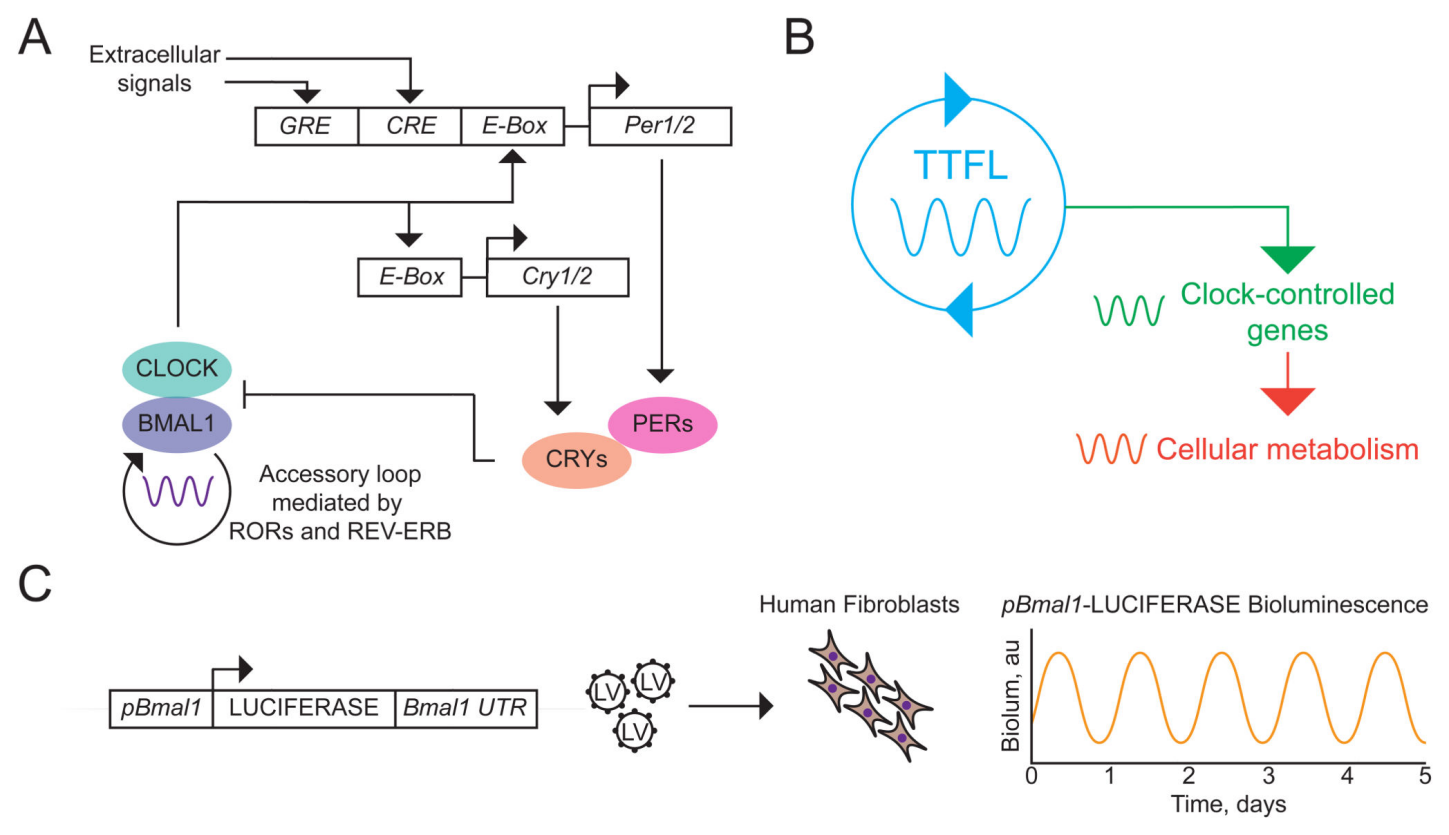
The cell-autonomous circadian clockwork. A. Schematic view of the transcriptional/translational negative feedback loop (TTFL) incorporating positive (CLOCK:BMAL1 proteins) and negative (PER:CRY proteins) regulators that oppose their transactivation by CLOCK:BMAL1 at E-box regulatory sequences. The core loop is stabilised by an accessory loop controlling *Bmal1* expression, and its phase is regulated by signalling cascades that converge on glucocorticoid (GRE) and calcium-cAMP (CRE) regulatory elements, especially in the *Per* genes. B. The cell-autonomous TTFL (depicted in A) controls the circadian expression of clock-controlled genes (CCGs) that in-turn orchestrate circadian cycles of cellular metabolism. C. Demonstration of spontaneous circadian TTFL function in human fibroblasts by lentiviral (LV) transduction with a luciferase reporter based on the *Bmal1* promoter (left) and subsequent bioluminescent recording for several days (right). Based on [79].

The ability of the isolated SCN to sustain circadian cycles of metabolic and electrical activity begs the question of the identity of the autonomous clock mechanism in mammals. The Noble Prize-winning answer come from genetic analysis, first in fruit-flies and then in mice [61]. By employing forward-genetic mutagenesis and comparative homology screens, the cellular clock has been identified as a transcriptional/post-translational feedback loop (TTFL), in which expression of the *Period* (*Per1*, *Per2*) and *Cryptochrome* (*Cry1*, *Cry2*) genes is trans-activated by heterodimers of the positive regulators CLOCK and BMAL1 [62] (Figure 3A). The encoded PER and CRY proteins act as negative regulators to oppose the actions of CLOCK:BMAL1, blocking their ability to transactivate their target E-box DNA sites in the *Per* and *Cry* genes. Although transactivation marks the start of circadian day, the synthesis and nuclear accumulation of PER and CRY takes about 12 hours, thereby delaying the onset of negative feedback that leads to oscillation. Moreover, it takes ~12 hours for PER and CRY to be degraded before transactivation can start again, leading to an overall period of ~24 hours. The alternation between transcriptional activation and suppression via E-box regulatory sequences also drives the rhythmic expression of a large number of clock-controlled genes (CCGs) that also carry E-boxes. These act as the output of the TTFL to control cellular circadian functions, not least the metabolic and electrical activity rhythms of the SCN (Figure 3B). This core loop is augmented by additional negative and positive feedback mediated by several nuclear receptors (Rev-erbα and β, RORα), the expression of which is driven by the core loop and which inhibit and activate the expression of *Bmal1*, respectively [62]. This interlocking further stabilises the TTFL, and mutations of these factors lead to changes in period or loss of rhythmicity altogether. Furthermore, the transcriptional activity of these rhythmically expressed nuclear receptors imposes circadian rhythmicity over the expression of a further large cohort of CCGs, many of which control cellular metabolism [63] and so augment circadian co-ordination. In turn, metabolic state is signalled back to the TTFL via chromatin modifications at the promoters of CCGs, further enhancing temporal integration and stability, as output again becomes input to the system [64]. Importantly, the TTFL mechanism is highly conserved across mammals, such that experimental or spontaneous mutations that stabilise or destabilise PER and CRY proteins can, respectively, lengthen or shorten the intrinsic period of the SCN and the dependent SWC of both experimental rodents and humans [46] [65]. For example, destabilisation of PER proteins can accelerate the mouse SCN clock by up to 4 hours [66], whereas stabilisation of CRY proteins can slow it down by 3 hours [67], without affecting the robustness or precision of the clock, whilst comparable mutations in humans are associated with advanced and delayed sleeping patterns [68, 69].

The elucidation of the SCN TTFL was followed by an even more remarkable discovery: that the cellular TTFL is active in almost all major organs on the body [70]. This was first apparent in time-series studies of *Per* gene expression levels in tissues, but the analysis was refined by the development of reporter genes, in which elements of the Per, *Bmal1* and *Cry* genes were modified to express bioluminescent and fluorescent proteins [71–74] (Figure 3C). When cultured ex vivo, heart, lung, liver, brain sub-regions, skin etc., exhibit spontaneous rhythms of TTFL-based gene expression. This over-turned the conventional view that the SCN was THE circadian clock, and made it possible to talk about a circadian network of interacting clocks. The role of the SCN is not as a primary driver to this network, but as a synchroniser and co-ordinator of the autonomous clocks within the network. In the absence of the SCN, the network rapidly loses coherence as local clocks drift apart and lose amplitude. Furthermore, the SCN is the only part of the network that receives entraining cues from the retina and so its phase determines that of the network, matching it to solar time. This is accomplished by the glutamatergic activation of electrical firing and Ca^2+^-dependent *Per* gene expression in the SCN [75], the elevation of *Per* delaying the TTFL at dusk and advancing it when it occurs around dawn [76].

The discovery of this circadian clock network revealed the mechanistic basis for the pervasive circadian control of metabolism and physiology: local clocks drive local cycles of gene expression that direct tissue-specific functions, and these are co-ordinated in time by the SCN. As with so much else of circadian organisation, the general principle of a distributed clock network revealed in experimental animals can be translated directly to humans. Serial skin biopsies exhibit daily cycles of clock gene expression in volunteers [77], and more recent post-mortem studies have revealed that ~50% of protein-coding genes cycle in at least 1 of 13 human tissues analysed [78]. Furthermore, cultured human fibroblasts or keratinocytes, transformed with virally encoded bioluminescent reporters, display robust cycles of TTFL activity with periods of ~24.5 [79], closely matching the average of human physiological rhythms [1] (Figure 3C). The cell-autonomous clock is therefore likely just as prevalent across tissues as observed in other species.

### 4 SCN control of the peripheral clock network

The discovery of a distributed clock network across all tissues immediately begs the question of how does the SCN orchestrate it to maintain a coherent internal temporal programme? The answer lies in the ability of the SCN to control a series of inter-dependent and partly redundant neural, endocrine and behavioural pathways (Figure 2A). The best mapped neural pathway controls the synthesis and release of melatonin by the pineal gland, which is a robust marker of circadian night in diurnal and nocturnal species alike, and is commonly used as a phase marker in human studies. A multi-synaptic pathway from the SCN via the hypothalamic paraventricular nucleus (PVN) and pre-sympathetic neurons of the spinal cord conveys circadian cues that direct the nocturnal stimulation of pinealocytes by their sympathetic innervation [80]. In seasonal mammals, changes in the duration of the nocturnal melatonin peak signal daylength to pituitary and hypothalamic sites expressing G-protein coupled melatonin receptors to coordinate species-specific, annual reproductive and metabolic cycles [81]. Although evolution and modern life-styles have attenuated seasonality in humans, melatonin nevertheless has residual activities, for example, exogenous melatonin can be used to reduce core body temperature, improve sleep quality [1] and to stabilise a disrupted SWC [34]. Furthermore, disruption of the circadian regulation of endogenous melatonin secretion is a common feature of ageing and neurodegenerative disease [82], with the potential to disrupt circadian coherence further. Parallel to melatonin, circadian control over core body temperature (CBT) is also mediated by SCN efferents running via the PVN and sub-paraventricular zone (SPZ) of the hypothalamus [83, 84]. Consequently, CBT cycles across the SWC but is generated independently of it, persisting under Constant Routine studies [1]. Importantly, the range of temperatures observed across the circadian cycle is sufficient to entrain the TTFL of mammalian cells, highlighting the SCN-dependent CBT rhythm as an important internal synchroniser in humans and other species [85, 86]. Consistent with its role as principal pacemaker, however, the SCN is not responsive to temperature cycles: the broadcast of phase information is unidirectional from SCN to the periphery [85]. Other neural pathways from the SCN to arousal centres in the hypothalamus and brain stem convey circadian cues to regulate the SWC [87], for which an intact TTFL in the SCN is both necessary and sufficient [88]. The end-point effector

pathways, such as the hypocretin arousal system of the dorso-lateral hypothalamus, have equivalent roles in both diurnally (human) and nocturnally active (mice) species [89]. What is not clear, however, is the point in the intervening circuitry at which the common SCN circadian signal is converted to species-specific activity patterns.

Regardless of activity phase, wakefulness is preceded by the secretion of corticosteroid hormones that prepare the body for physical activity, with a peak around dawn in humans and around dusk in nocturnal rodents. This circadian control is mediated by SCN projections to PVN neurons [84, 90] that in turn release corticotrophin-releasing hormone (CRH) at the median eminence to drive daily rhythms of circulating adrenocorticotrophin (ACTH) [91]. Corticosteroid receptors are widely and abundantly expressed across tissues and so the resulting rhythm of circulating cortisol in humans and corticosterone in rodents is an effective broadcast of circadian time. Indeed, synthetic corticosteroids have a powerful resetting effect on local clocks, phase-shifting the TTFL and synchronising the local transcriptome both in culture and in vivo [91–93]. In adrenalectomized rodents, peripheral tissues remain rhythmic, but their TTFL moves to atypical phases, an effect that can be mitigated by treatment with corticosteroid [94]. Moreover, their phase is more labile than in intact animals, highlighting the role of corticosteroids as a “temporal anchor”. The mechanisms whereby corticosteroids synchronise cellular TTFLs are likely very diverse, given the broad transcriptional activity of corticosteroid receptors, although the most direct routes are via glucocorticoid response elements (GREs) present in the *Per1* and *Bmal1* genes [93, 95], as well as downstream clock-controlled transcription factors [96]. Importantly, the absence of glucocorticoid receptors from the SCN ensures that, as with CBT, the transfer of phase information is unidirectional: the SCN sets the phase of the temporal anchor but not vice versa. Given their pivotal role in internal synchronisation across tissues, disrupted corticosteroid rhythms in, for example, shift workers will carry a heavy metabolic and/ or psychiatric burden. Furthermore, the precision and amplitude of the corticosteroid rhythm, and therefore its ability to maintain circadian coherence, decline with ageing, so adding to morbidity. Conversely, interventions targetting the corticosteroid rhythm, as a circadian intermediary between the SCN and peripheral metabolism, may have therapeutic value in a variety of conditions [91].

Finally, perhaps the most pervasive and powerful internal synchroniser is a metabolic consequence of the SWC: the feeding and fasting cycle (FFC). This indirect product of SCN time-keeping is normally entrained to solar time but the experimental manipulation of food availability can have powerful effects on patterns of behaviour, masking its SCN-dependent control by elevating activity in anticipation of scheduled food presentation [97]. The neural circuits underlying food anticipatory activity (FAA) are not known but may involve the reward circuits of the brain. They certainly do not require an anatomically or genetically intact SCN [97, 98]. Ordinarily, feeding is aligned with the spontaneous SWC, but when food availability is scheduled to other times, the altered behavioural patterns are accompanied by re-phasing of the TTFL of most peripheral organs, most obviously the liver [99]. When monitored with bioluminescent TTFL reporters in ex vivo culture or in vivo, the liver clocks of rats and mice subjected to restricted feeding in daytime are advanced by ~10 hours within 2 days, even though the SCN remains phase-locked to the lighting schedule (or has been ablated) [100, 101]. Accompanying this advance of the TTFL are significant changes in the circadian transcriptome and metabolome of the liver, which is controlled directly by both the cell-autonomous clock and indirectly by feeding-related cues [102]. Inevitably, numerous circulating cues are associated with the FFC. In cultured cells and tissues ex vjvo, treatment with serum, which contains numerous active factors such as hormones, nutrients and metabolites, and can reset the cell-autonomous TTFL. The most direct route for this is via GREs and calcium-response elements (CREs) in the *Per* genes. More specific to the FFC, it is also clear that insulin and IGF-1, both induced by feeding, can induce PER protein levels and thereby entrain the TTFL of peripheral tissues to feeding time [103]. Although elucidated by manipulations in experimental rodents, the principle that the FFC is a critical internal synchronising factor in humans is now clear. Furthermore, it informs understanding of the detrimental effects of shift-work, with altered meal timings, on metabolic health: behaviourally induced temporal mis-alignment of anabolic and catabolic processes may well be the underlying cause of elevated cardiovascular and metabolic diseases, including type II diabetes, in life-time shift-workers [104]. Equally, such knowledge provides potential ways to mitigate these effects [29].

### 5 What makes the SCN such a powerful clock?

**Figure 4:**
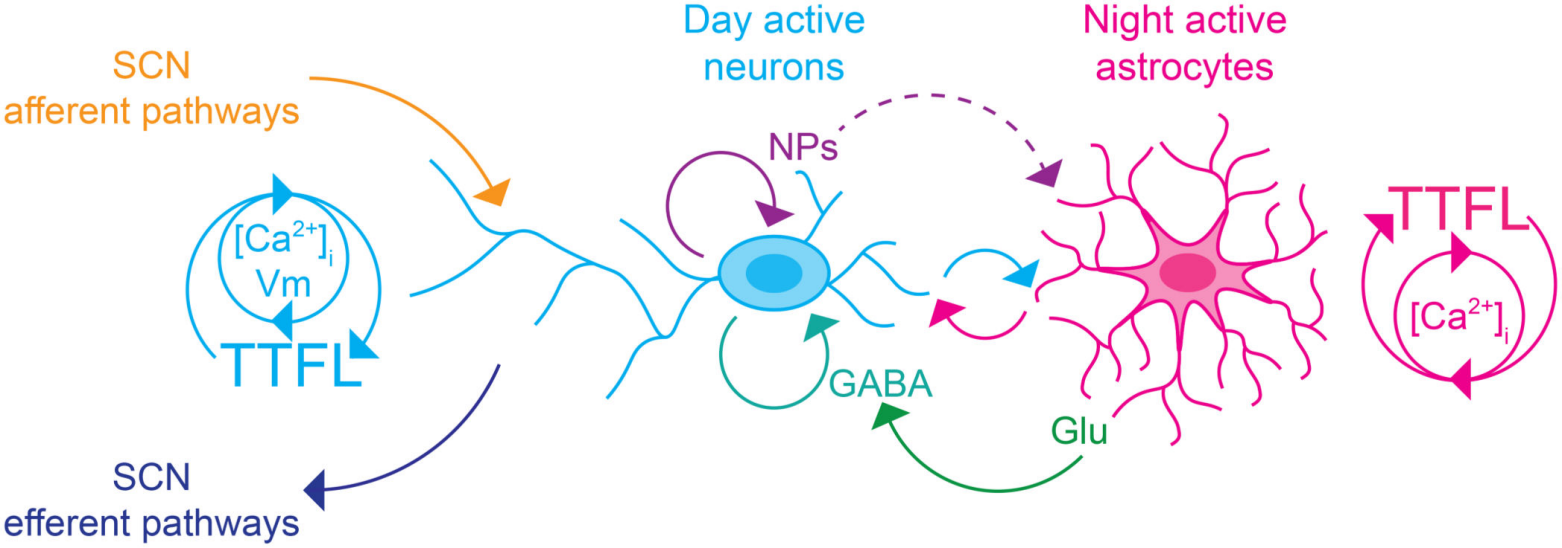
Interactions between neurons and astrocytes drive circadian time-keeping in the SCN. Both neurons (blue) and astrocytes (magenta) contain a TTFL but their cellular activity rhythms, as evidenced by rhythms of calcium ([Ca^2+^]_i_), are oppositely phased (neurons day-, astrocytes night-active). Neuropeptides (NPs) and GABA synchronize the SCN neuronal network, and astrocytes signal via glutamate (Glu) (and likely other astrocyte-derived signals, magenta arrow) to regulate the neuronal rhythms. Equally, neuronal cues (yet to be identified, blue, and possibly including neuropeptides, broken line) signal circadian information to astrocytes. This reciprocal communication enhances circuit-level time-keeping. Afferent signals onto neurons from outside the SCN determine network phase, and neuronal efferents broadcast circadian time to SCN targets in the brain.

To function as well as it does, day to day, year to year, the circadian network requires robust and precise signals of high-amplitude from the SCN. The circadian cycles of electrical activity and TTFL gene expression observed in the SCN offer this, but what are the mechanisms that achieve it? At the cell-autonomous level, the TTFL incorporates several interdependent feedback loops, which will provide mutual reinforcement [105], but this is also the case in peripheral tissues, so there must be something more. Significantly, the electrical activity of SCN neurons feeds in to the TTFL, insofar as the expression of *Per* genes is driven by daytime elevation of intracellular calcium ([Ca^2+^]_i_), which acts through CREs in the *Per* genes to drive their expression, amplifying the effect of the E-boxes (Figure 4). Thus, an output of the TTFL becomes a stimulatory input to it, adding amplitude and robustness [75]. Beyond this cell-autonomous relationship, however, is the network-level organisation of the SCN, such that electrical activity in some neurons will drive the electrical activity of their target cells. When electrical synaptic signalling is compromised pharmacologically [106, 107] or genetically [108], or the network is physically disrupted in dispersed culture [109], cellular TTFLs are unable to synchronise and lose amplitude and coherence. Intercellular coupling is therefore a critical property of the SCN time-keeper, and the most obvious coupling factors are the various neuropeptides expressed by distinct cell populations of the SCN [40, 46] (Figure 2B). Beyond the core/shell dichotomy of the SCN lies a more complex neuropeptidergic topology, centred around signalling axes consisting of distinct populations that express a ligand and other cells the cognate receptor for the ligand [44, 45]. Such ligand-receptor axes, involving VIP, AVP or PROK2 signalling, are essential for circuit-level coherence of the SCN and are able to act as pace-making hubs that determine ensemble period and phase [45, 108, 110, 111]. This is sustained by coupling of the receptors to *Per* gene expression [112], most directly via CREs [113, 114]. In the absence of neuropeptidergic coupling in genetically modified mice, the SCN cannot keep effective time and circadian control over behaviour and physiology is compromised. The presence of the same neuropeptides and receptors indicates that similar mechanisms apply in the human SCN, and their decline may contribute to loss of circadian amplitude and coherence in older people [115].

Until recently, the powerful synchronising effects of electrical activity and neuropeptides focussed attention on the role of neurons in conferring robustness, amplitude and resilience to SCN time-keeping. Indeed, given that the electrical firing rhythm of SCN neurons conveys its circadian output signal [108, 116], they are the ultimate arbiters of circadian behaviour and physiology. Nevertheless, the SCN also contains abundant astrocytes and they also contain active TTFLs [117, 118] (Figure 4). Surprisingly, these run almost in antiphase to the neuronal TTFL and this is also evident in the circadian cycle of [Ca^2+^]_i_, which peaks at night whereas neuronal [Ca^2+^]_i_ peaks in circadian day [117]. The wider SCN circuit therefore consist of day-active neurons and night-active astrocytes, which suggests some reciprocally inhibitory relationship, or at least an important temporal coupling. But is the cell-autonomous clock of SCN astrocytes important for circadian behaviour? Indeed, it is. When the intrinsic period of the astrocyte TTFL is lengthened by cell-type-specific deletion of the *Tau* allele of casein kinase 1 epsilon (*CK1e^Tau^*) which shortens TTFL period by destabilising PER proteins [66], the period of circadian behavioural rhythms is lengthened [117, 118]. This demonstrates that SCN astrocytes are able to impose their cell-autonomous period onto the SCN network and thereby control the neuronal signals conveyed to SCN targets in the brain.

The potency of this astrocyte-to-neuron circadian signalling is apparent in SCN that lack CRY proteins and are therefore arrhythmic. Selective expression of CRY1 in neurons to initiate their cell-autonomous rhythms restores TTFL function across the circuit, as would be expected, but equally so does CRY1 complementation solely in astrocytes [119, 120]. Furthermore, not only do the CRY1-complemented astrocytes initiate rhythms in neuronal [Ca^2+^]_i_, but they also do so with a long period specific to a CRY1-driven TTFL. Temporal information encoded by SCN astrocytes is therefore conveyed to “clockless” SCN neurons, and ultimately drives circadian behaviour. There are, however, two notable differences between SCN neurons and astrocytes in their circadian competence. First, changes in the cellular activity of neurons but not astrocytes can change the phase of the SCN. This is consistent with the innervation of SCN neurons by the RHT and afferents from the brain stem that deliver resetting cues [120] (Figure 4). Astrocytes do not receive information regarding external time and so are not likely to have the capacity to determine SCN phase. Second, in initiating SCN rhythms de novo and changing their period, astrocytes take longer than do neurons. This is consistent with the view that SCN astrocytes regulate neuronal function by indirect mechanisms, whereas neurons have immediate access to their synaptically wired networks. One potential signalling mechanism involves extracellular glutamate ([Glu^-^]_e_), which is highly circadian in SCN slices and, given that SCN neurons are GABAergic, likely comes from astrocytes. A function for [Glu^-^]_e_ as a circadian gliotransmitter is underlined by its rhythm: it peaks at night with a profile that maps directly onto the rhythm of astrocytic [Ca^2+^]_i_ and disruption of the rhythm by blockade of glutamate uptake disrupts the SCN oscillation. In addition, neurons in the dorsal SCN express NMDA receptors with a specific sub-unit, NR2C (Figure 2B) and pharmacological blockade of NR2C disrupts SCN timekeeping, suppressing the TTFL and desynchronising cellular oscillators [117]. How the nocturnal elevation of [Glu^-^]_e_ inhibits SCN neurons, and how SCN neurons communicate circadian time to astrocytes remain unclear. Nevertheless, the reciprocal, mutually reinforcing actions of these two populations of clock cells will add further robustness, amplitude and accuracy to the ongoing SCN oscillation.

### 6 Summary and prospect: clocks and neurodegenerative disease

**Figure 5:**
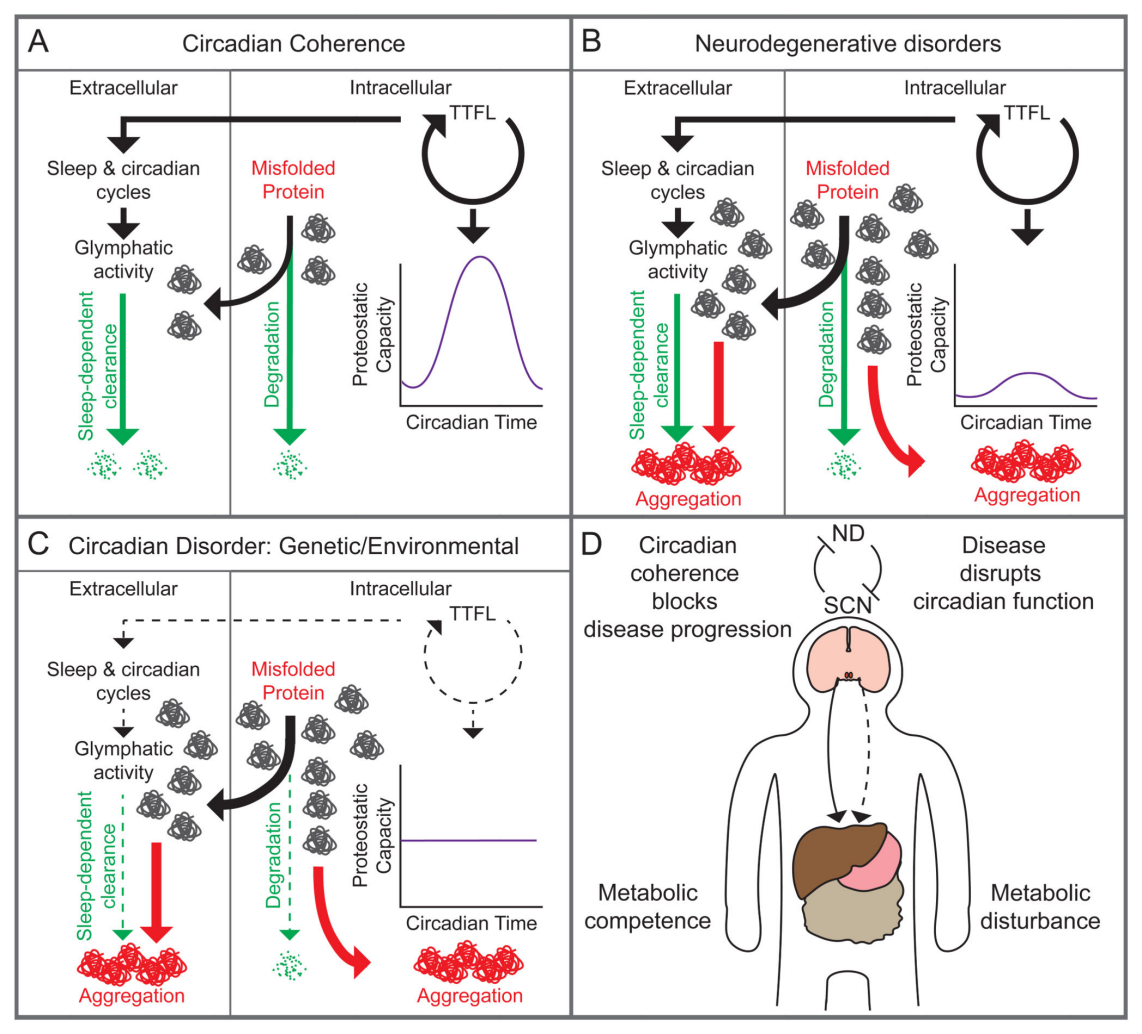
Putative reciprocal links between the circadian system and neurodegenerative disease. A. With circadian coherence, intra-cellular proteostatic capacity is regulated in time by the TTFL, degrading unwanted proteins efficiently and in sequence with other cellular processes (right), whilst sleep-dependent glymphatic activity efficiently clears extracellular proteins (left). B. The balance is lost in neurodegeneration when disease-causing factors (age- and lifestyle-related, mutations to proteins and their metabolic pathways), overwhelm the capacity of the circadian- and/ or sleep-regulated systems. C. This balance is also lost when the circadian system and/ or sleep are compromised due to genetic, environmental or age-related factors, increasing the potential for intra- and/ or extra-cellular aggregation. D. At a systems level, circadian coherence in the SCN suppresses neurodegenerative (ND) disease progression and sustains metabolic competence in the periphery (solid line). Conversely, ND progression can reduce circadian competence. Consequently, with loss of circadian competence ND will progress, and further compromise the SCN clock and/ or sleep, creating a downward spiral. This will further compromise circadian regulation of other systems in peripheral organs (broken line), leading to metabolic disturbance.

In the context of neurodegenerative diseases, such as Huntington’s disease, that are characterised by the accumulation of intracellular and/or extracellular protein aggregates, there is growing awareness of potential reciprocal interactions between the circadian system and disease progression (Figure 5). Most obviously, neuronal loss during neurodegeneration may compromise clock- and sleep-relevant circuits and thereby have a negative impact on quality of life, brain function and physiological resilience [5]. Furthermore, loss of circadian coordination in peripheral tissues, as observed in animal models of disease [121], may have metabolic consequences, for example contributing to disease-related cachexia [122]. Given the intimate relationship between glial cells and neuronal function, including sleep and time-keeping, the marked inflammatory responses by microglia and astrocytes that are associated with neurodegenerative disease may further disrupt sleep/clock mechanisms [123]. Conversely, given that the cell-autonomous clock controls all aspects of cellular homeostasis, including redox state and inflammation [124], disease-dependent impairment of cellular circadian time-keeping may further exacerbate disease progression by mis-regulation of these states, locking disease and the clock into a self-reinforcing downward spiral. A similar scenario applies to the circadian regulation of protein quality control systems. With coherent time-keeping, over-expressed, damaged and misfolded proteins will be degraded, recycled and/or cleared in a timely, sequenced manner. With a disrupted clock, however, the inter-dependent clearance systems will become less effective and such proteins may accumulate, tipping the balance towards aggregation. Furthermore, sleep facilitates the clearance of brain metabolites, including extracellular amyloid beta, via glymphatic and other pathways [125, 126], and so disrupted sleep or circadian time-keeping may compromise this and thereby exacerbate disease progression [127]. Overall, therefore, a two-way linkage exists between sleep and clocks on the one hand and neurodegenerative conditions on the other [6, 127]. Importantly, better understanding of these relationships offers the potential to use the maintenance of sleep and circadian coherence as means to delay disease progression and mitigate its effects on the quality of life of patients.
